# Inbreeding and pedigree analysis of the European red dairy cattle

**DOI:** 10.1186/s12711-022-00761-3

**Published:** 2022-10-23

**Authors:** Sofia Nyman, Anna M. Johansson, Valentina Palucci, Anna A. Schönherz, Bernt Guldbrandtsen, Dirk Hinrichs, Dirk-Jan de Koning

**Affiliations:** 1 Department of Animal Breeding and Genetics, Swedish University of Agricultural Sciences, Uppsala, Sweden; 2 Interbull Centre, Department of Animal Breeding and Genetics, Swedish University of Agricultural Sciences, Uppsala, Sweden; 3 Department of Animal Science, Aarhus University, Tjele, Denmark; 4 Department of Veterinary and Animal Sciences, University of Copenhagen, Copenhagen, Denmark; 5 Department of Animal Breeding, University of Kassel, Witzenhausen, Germany

## Abstract

**Background:**

Red dairy cattle breeds have an important role in the European dairy sector because of their functional characteristics and good health. Extensive pedigree information is available for these breeds and provides a unique opportunity to examine their population structure, such as effective population size, depth of the pedigree, and effective number of founders and ancestors, and inbreeding levels. Animals with the highest genetic contributions were identified. Pedigree data included 9,073,403 animals that were born between 1900 and 2019 from Denmark, Finland, Germany, Latvia, Lithuania, the Netherlands, Norway, Poland, and Sweden, and covered 32 breeds. The numerically largest breeds were Red Dairy Cattle and Meuse-Rhine-Yssel.

**Results:**

The deepest average complete generation equivalent (9.39) was found for Red Dairy Cattle in 2017. Mean pedigree completeness ranged from 0.6 for Finncattle to 7.51 for Red Dairy Cattle. An effective population size of 166 animals was estimated for the total pedigree and ranged from 35 (Rotes Höhenvieh) to 226 (Red Dairy Cattle). Average generation intervals were between 5 and 7 years. The mean inbreeding coefficient for animals born between 1960 and 2018 was 1.5%, with the highest inbreeding coefficients observed for Traditional Angler (4.2%) and Rotes Höhenvieh (4.1%). The most influential animal was a Dutch Meuse-Rhine-Yssel bull born in 1960. The mean inbreeding level for animals born between 2016 and 2018 was 2% and highest for the Meuse-Rhine-Yssel (4.64%) and Rotes Hohenvieh breeds (3.80%).

**Conclusions:**

We provide the first detailed analysis of the genetic diversity and inbreeding levels of the European red dairy cattle breeds. Rotes Höhenvieh and Traditional Angler have high inbreeding levels and are either close to or below the minimal recommended effective population size, thus it is necessary to implement tools to monitor the selection process in order to control inbreeding in these breeds. Red Dairy Cattle, Vorderwälder, Swedish Polled and Hinterwälder hold more genetic diversity. Regarding the Meuse-Rhine-Yssel breed, given its decreased population size, increased inbreeding and low effective population size, we recommend implementation of a breeding program to prevent further loss in its genetic diversity.

## Background

The modern European red dairy cattle breeds are widely used both within and outside Europe. The European red dairy breeds are known for their functional characteristics and good health including a higher fertility, fewer claw and leg problems, excellent udder health, easy calving, and lower incidence of still birth compared to the Holstein–Friesian breed [1–4]. In addition, a considerable proportion of the modern red dairy breeds in Europe are good producers with high milk yields and high milk protein and fat levels.

Animal husbandry in Europe has historically been characterized by a large phenotypic variation between cattle populations from different geographical regions [5]. Because of the demands of high-input farming systems, local cattle breeds are almost totally replaced by commercial red dairy breeds and the Holstein–Friesian breed [5, 6]. Breed formation and selective breeding have created genetic subdivisions and reduced effective population sizes [7], which have led to the disappearance of a significant number of cattle breeds, to an increase in the number of endangered breeds, and more specifically to a decrease in the genetic diversity of the red dairy cattle breeds in Europe [6].

Maintaining the genetic diversity of breeds is crucial for future improvements of European livestock in order to respond to changes in climate, emerging diseases, or consumers’ preferences [8, 9]. The efficiency of modern breeding methods, such as genomic selection and reproductive technologies, which result in increased selection intensities, lead to the rapid propagation of particular desirable genotypes and hence to a loss in genetic diversity. Maintaining long-term sustainability of animal production systems and food security requires the conservation and management of livestock genetic resources [10–12]. Furthermore, the preservation of breeds that have unique genetic characteristics is important [13]. However, because genetic conservation programs are costly to implement and operate, it is not possible to conserve all the genetic variation in all populations. An essential first step is the evaluation of the genetic resources and the selection of appropriate populations for conservation. Then, it is necessary to implement a strategy that will maintain the highest possible level of genetic variation across the species, both within and among breeds [14]. Maintaining genetic variation within a breed is also important for its commercial future [8]. An increase in homozygosity within a breed can cause inbreeding depression and hence markedly reduce performance, particularly for fertility traits, and intensify the incidence of monogenic recessive disorders. In the Holstein population, the widespread use of a few elite sires has led to an increased incidence of genetic disorders such as BLAD (bovine leukocyte adhesion deficiency [15], MFD (mulefoot disease), CVM (complex vertebral malformation [16], DUMPS (uridine monophosphate synthase deficiency [17]), and others.

Pedigree analysis is an important tool to describe the genetic diversity of a breed and its evolution across generations [18]. Inbreeding results from non-random mating and genetic drift (reviewed in [19]) and changes in inbreeding levels are often used to estimate genetic drift. In dairy cattle populations, inbreeding is relatively common, especially in populations using modern selection practices, such as artificial insemination, where a small number of bulls are used extensively in breeding programs, which tends to increase the rate of inbreeding and reduce effective population sizes (reviewed in [6, 19]).

Another threat to the genetic integrity of dairy cattle breeds is the use of bulls from higher-yielding breeds as a shortcut to improve performance. Crossing and subsequent selection may erode the genetic composition of the recipient breed. Pedigrees contain (or should contain) information about such genetic introgression.

Animal numbers have been declining in many European red dairy breeds (e.g., [20, 21]) and to effectively manage the genetic diversity in this situation, a comprehensive knowledge of their pedigrees is essential. Conservation of the native red cattle breeds and increasing the competitiveness of the high-producing modern red dairy breeds are important for the survival of breeds. Based on pedigree records, our aims were to investigate the population structure, generation intervals, and inbreeding levels in European red dairy cattle breeds, to identify the ancestors that have made the greatest contributions to the pedigree, and to explore the gene flow between breeds.

## Methods

### Data

Pedigree information was collected from the Animal Breeders Association of Latvia (ABAL, Latvia), CRV (the Netherlands), Geno (Norway), Lithuanian Red Cattle Improvement Association (LRCIA, Lithuania), Viking Genetics (VG, Denmark, Sweden, Finland), Vereinigte Informationssysteme Tierhaltung w.V. (VIT, Germany), and Wroclaw University of Environmental and Life Sciences (WUELS, Poland). The submitted records included information on the animal, sire, and dam ID and birth dates. Pedigrees were created according to the Interbull international ID structure (Section 9—Dairy Cattle Genetic Evaluation, ICAR Guidelines [22]) and all the information was collected through a dedicated version of the Interbull Data Exchange Area (IDEA) database and underwent the same pedigree verification process as applied by Interbull Centre for any given international genetic evaluation. More information about the submission process is in Appendix.

The total number of unique animals in the resulting database was 9,073,403. Data on the number of animals in the pedigree for each breed and for each sex are in Table 1. Animals were born between the early 1900s and 2019, with a majority born after 1960. In this study, we used the birth year 1960 as the base year to have the largest possible number of animals with sufficient connectivity to make meaningful inferences. In total, 9,036,935 animals born between the 1960s and the end of 2018 were retained for further analyses.

**Table 1:** Characterization of the pedigree information

Breed	Number of animals in the database^e^	Number of females in the database^e^	Number of males in the database^e^	Number of animals born 1960–2018
Red Dairy Cattle (RDC)^a^	6,014,920	4,776,217	1,238,703	5,999,780
Meuse-Rhine-Yssel (MRY)^a^	2,498,187	2,238,659	259,528	2,496,714
Vorderwälder (VWD)^a^	228,051	216,884	11,167	226,744
Holstein (HOL)^b^	184,432	160,338	24,144	182,410
Swedish Polled (SKB)^a^	40,613	37,424	3184	39,059
Simmental (SIM)^b^	17,623	13,104	4519	15,240
Red Holstein (RED)^b^	10,807	9259	1548	10,755
Jersey (JER)^b^	10,434	8513	1922	10,205
Finncattle (FIC)^a^	4455	3703	752	4455
Braun Swiss (BSW)^b^	2339	1628	711	2149
Hinterwälder (HWD)^a^	1135	756	370	1040
Traditional Angler (RVA)^a^	1079	990	89	1053
Rotes Hohenvieh (RHV)^a^	756	553	203	749
Other breeds^c^	58,572	56,087	2448	58,385
ALL^d^	9,073,403	7,524,115	1,549,288	9,036,935

^a^Breeds with more than 500 animals born between 1900 and 2019

^b^Non-targeted breeds not included in the analysis

^c^Breeds with less than 500 animals that were not included in the analysis, i.e. British Friesian, Dutch Friesian, Simmental dual-purpose, Belgian Blue, Pinzgauer, Charolais, Angus, Aubrac, Blonde d’Aquitaine, Tyrol Grey, Hereford, Tux, Belgian White and Red, Limpburger Cattle, Bohuskulla, Galloway, Montbelliard, Limousin and crosses

^d^ALL includes all the breeds in the database

^e^Animals born between 1900 and 2018

The country of origin and breed code for each animal were derived from the international animal ID. The total number of countries of origin was 30 and the number of registered breed codes was 32. The breeds were defined according to the breed codes assigned by the Interbull Centre (The Interbull Centre, 2020). Some of these breed codes are collective codes for several national breeds, e.g., Red Dairy Cattle (RDC), which includes Swedish Red, Danish Red, Finnish Ayrshire, Polish Red, Norwegian Red, Lithuanian Red and Latvian Brown among others. The Swedish polled (SKB) includes the Swedish native breeds: Swedish Polled, Swedish Mountain Cattle and Swedish Red Polled. Finncattle (FIC) includes the Finnish native breeds: Eastern, Western and Northern Finncattle. In the current study, some national breeds were submitted to the pedigree with their national breed codes, e.g., Rotes Höhenvieh (RVH), Traditional Angler (RVA), Vorderwälder (VWD) and Hinderwälder (HWD). The two numerically largest breeds were RDC with 6,014,920 animals and Meuse-Rhine-Yssel (MRY) with 2,498,187 animals (Table 1).

The analyses in the current study focus on the red dairy cattle breeds in Europe and on related local breeds. Other breeds including Holstein (HOL), Simmental (SIM) and Aberdeen Angus were also present in the pedigree, either as parents of a targeted red dairy animal that had been crossed for improved milk production, or for dual-purpose breeding. Data from the non-targeted breeds are not representative for the red dairy cattle breed and were excluded from further analyses, mainly because of their high level of cross breeding and of their low pedigree completeness level. Unless otherwise stated, the populations used in the analyses included the targeted red dairy breeds (RDC, MRY, VWD, SKB, FIC, HWD, RVA and RHV) with more than 500 animals in the pedigree, born between 1960 and 2018.

### Pedigree analysis

We used the software package RelaX2 [23] for pedigree analyses and although the methodology is described at the population level, the procedures were the same at the breed level, unless otherwise stated. Illustrations were prepared in R version 3.6.1 [24].

### Pedigree completeness

#### Equivalent complete generations

One way to characterize pedigree data is to estimate the number of complete generation-equivalents, defined as the sum of the proportion of known ancestors over all traced generations. The equivalent complete generation (ECG) was estimated as the sum of ${(1/2)}^{n}$, where $n$ is the number of generations separating an individual to each known ancestor [25]. Equivalent complete generations for animal $i$ is:

$${t}_{i}=\sum\limits_{j=1}^{{k}_{i}}{\left(1/2\right)}^{{n}_{j}},$$

where ${k}_{i}$ is the number of ancestors for animal $i$ and ${n}_{j}$ is the number of generations between animal $i$ and its ancestor $j$.

### Subpopulations

Subpopulations were identified within the entire database. The definition of a subpopulation is a group of animals that are connected via a common ancestor or via descendants [26]. Animals that are in different subpopulations have no known common ancestors or descendants in the pedigree. Subpopulations can represent missing pedigrees as well as separate genetic groups.

### Generation intervals

Generation intervals were calculated along the four gametic pathways: sire to son (${l}_{ss}$), sire to daughter (${l}_{sd}$), dam to son (${l}_{ds}$) and dam to daughter (${l}_{dd}$). The four generation intervals were based on recorded birth dates of animals together with those of their sires and dams. The average generation interval ($\overline{l }$) was computed by:

$$\overline{l } = \frac{{l}_{ss}+ {l}_{sd} + {l}_{ds} + {l}_{dd}}{4}.$$

### Effective population size

The effective population size (${\mathrm{N}}_{e}$) is the number of animals that would lead to the observed increase in inbreeding if each animal in the parent generation has an equal probability of being a parent to each animal in the filial generation [27]. The effective population size was estimated, based on [27] as:

$${\mathrm{N}}_{e}= \frac{1}{2 \overline{\Delta \mathrm{F}}},$$

where $\overline{\Delta \mathrm{F}}$ is the mean of the individual increase in inbreeding coefficient (${\Delta F}_{i}$) values, with ${\Delta F}_{i}$ being calculated as follows:

$${\Delta F}_{i}=1-(1-{F}_{i}{)}^{\frac{1}{{t}_{i}-1}},$$

where ${t}_{i}$ is the sum over all known ancestors of the term of ${(1/2)}^{n}$, $n$ being the number of generations separating the individual from each known ancestor.

### Effective number of founders

Founder animals (animals with unknown parents) are assumed to be unrelated and have an inbreeding coefficient of 0. The effective number of founders (${f}_{e}$) is the number of equally contributing founders that would be expected to produce a level of genetic diversity identical to that observed in the population under study [28, 29] and was estimated as:

$${{f}_{e}}={}^{1}/{}_{\mathop{\sum }_{i=2}^{{{N}_{f}}}q_{i}^{2}},$$

where ${N}_{f}$ is the number of founder animals, and ${q}_{i}$ is the expected genetic contribution of founder $i$ to the gene pool of the population (see Appendix A in Boichard et al. [30]). The expected contribution ${q}_{i}$ corresponds to the probability that a randomly sampled gene in this population originates from founder $i$.

### Marginal contributions

Marginal contributions, i.e., the contribution not yet explained by the other ancestors, were computed to account for relationships among ancestors by selecting the animal with the largest contribution to the population, then iteratively selecting animals with the largest contribution not accounted for by previously selected individuals. Marginal contributions (${p}_{k}$) were estimated as proposed by Boichard et al. [30] as:

$${p}_{k}={q}_{k}\left(1-\sum_{i=1}^{n-1}{a}_{i}\right),$$

where ${q}_{k}$ is the expected contribution and ${a}_{i}$ is the expected genetic contribution of the $i$th of the $n-1$ selected ancestors to individual $k$. The algorithm is presented in detail in Appendix B of Boichard et al. [30].

### Effective number of ancestors

The effective number of founders does not account for bottlenecks and strong selection in a pedigree [23, 29]. For this reason, the effective numbers of ancestors (${f}_{a}$), which is the minimum number of ancestors (founders and non-founders) needed to explain the complete genetic diversity of the population was estimated as:

$${{f}_{a}}={}^{1}/{}_{\mathop{\sum }_{k=1}^{{{N}_{a}}}p_{k}^{2}},$$

where ${p}_{k}$ is the marginal contribution of animal $k$, and ${N}_{a}$ is the number of animals with non-zero ${p}_{k}$ or the number of the most influential contributors. The ratio between the effective number of founders and the effective number of ancestors can be used as an indicator of a population bottleneck although strong selection can affect this ratio.

### Inbreeding coefficients and mean relationships

Inbreeding coefficients ($F$) were calculated by a modified version of the algorithm of Meuwissen and Luo [31]. Average inbreeding coefficients were estimated by birth year to determine the trends in inbreeding over time. The mean relationship was estimated as the average pairwise relationship coefficients between the animals within the same breed.

#### Purebred animals

Purebred animals were defined as animals with a breed proportion greater than 80%. To investigate the distribution of the level of inbreeding in purebred animals, they were classified according to their inbreeding levels and assigned to one of five groups; $F$ = 0; 0 < $F$  ≤ 0.0625; 0.0625 < $F$  ≤ 0.125; 0.125 < $F$  ≤ 0.25; or $F$ > 0.25.

The amount of Holstein ancestry was estimated across and within breeds by the expected proportion of genes that could be traced back to founders with an Interbull breed code of “HOL” (Black Holstein) or “RED” (Red Holstein).

#### Partial inbreeding

The proportion of $F$, i.e. the partial inbreeding coefficient ($pF$), was computed using the R-package gRain [32] for the 20 ancestors with the greatest marginal contributions to inbreeding of animals in the RDC populations born between 1960 and 2018 and between 2010 and 2018 (reviewed in [33]). Partial inbreeding estimates the contribution of one individual to the inbreeding level of the current population.

### Gene flow

Gene flow between breeds and between countries of origin was studied in animals born between 2010 and 2018. This was performed by studying the breed of the animals born between 2010 and 2018, which belonged to the targeted red dairy breeds, and the breed of the parents, which could belong to any breed. The results were illustrated by an alluvial plot produced using the R-package “ggalluvial-package” version 0.11.1 [34] in R [24]. Gene flow between countries, which occurs when a descendant has a parent with a different country code, was studied. Descendants were included if they were born in one of the countries included in the current study (Denmark, Finland, Germany, Latvia, Lithuania, the Netherlands, Norway, Poland and Sweden). Parents could be born in any country.

## Results

The number of animals born between 1960 and 2018 was 9,036,935. At the population level, the number of individuals with unknown parents was 1,559,777. Data on the population structure including ${\mathrm{N}}_{e}$, effective number of founders and ancestors, and mean inbreeding and relationship are in Table 2. The effective number of ancestors (founders or not) in the whole population, which is required to explain the genetic variability, was 165 and the effective number of founders was 349. The effective number of founders and ancestors was smallest for the MRY (74 and 50), RVA (72 and 62) and RHV (35 and 30) breeds.

**Table 2:** Effective population size (${\mathrm{N}}_{e}$), effective number of founders (${f}_{e}$) and ancestors (${f}_{a}$), mean inbreeding level, and mean relationship for the Red Dairy Cattle (RDC), Meuse-Rhine-Yssel (MRY), Vorderwälder (VWD), Swedish Polled (SKB), Finncattle (FIC), Traditional Angler (RVA), Rotes Höhenvieh (RHV), Hinterwälder (HWD) and the whole population (ALL) born between 1960 and 2018

Breed	Number of animals	${\mathrm{N}}_{e}$	${f}_{e}$	${f}_{a}$	Mean $F$ (%)	Mean relationship (%)
RDC	5,999,780	226	371	105	1.6	1.9
MRY	2,496,714	109	74	50	1.3	1.6
FIC	4455	85	668	670	–	–
SKB	39,059	166	240	76	1.0	1.4
VWD	226,744	178	282	131	1.0	1.6
HWD	1040	182	168	120	0.7	0.9
RVA	1053	64	72	62	4.2	4.3
RHV	749	35	35	30	4.1	4.5
ALL^a^	9,036,935	166	349	165	1.5	1.0

^a^ALL includes all the animals in the pedigree born between 1960 and 2018

Regarding the subpopulations, the largest one included 8,926,186 animals from all 30 countries of origin and from all 32 breeds (67.3% RDC, 27.9% MRY and 5% other breeds). Seven of the other subpopulations included more than 100 individuals and several subpopulations included only one breed originating from one country.

### Pedigree completeness

**Fig. 1:**
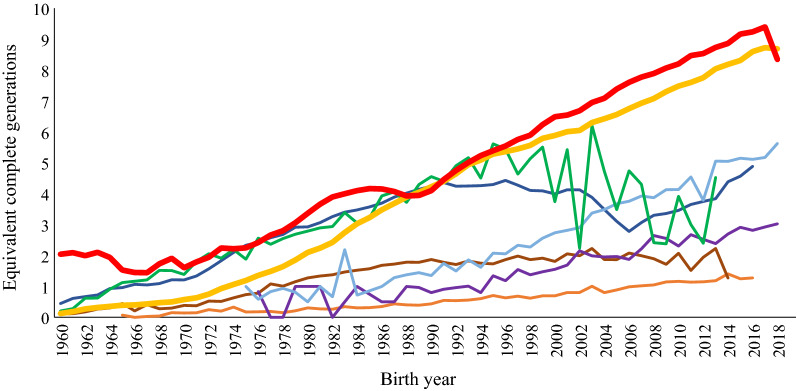
Complete generation equivalents by year of birth for Red Dairy Cattle (RDC; red line), Meuse-Rhine-Yssel (MRY; yellow line), Finncattle (FIC; orange line), Swedish Polled (SKB; brown line), Vorderwälder (VWD; dark blue line), Hinterwälder (HWD; green line), Traditional Angler (RVA; purple line) and Rotes Höhenvieh (RHV; light blue line) breeds over years

Complete generation equivalents by year of birth are shown in Fig. 1. Although the absolute value varied, ECG generally increased between 1960 and 2018, except for the HWD and VWD breeds. In 2017, the RDC and MRY breeds had the deepest pedigrees with an ECG of 9.24 and 8.72, respectively.

### Inbreeding and breed proportions

Sixty-eight percent of the 2,233,059 animals born between 2010 and 2018 were purebred animals (i.e. breed proportion of one breed higher than 80%). The breed purity and inbreeding level of the observed purebred breeds are in Table 3. The proportion of purebred animals was high in both MRY (99.6%) and SKB (95.7%), and lower in RDC (67.2%), VWD (75.0%) and HWD (36.8%). The proportion of purebred animals was low and particularly low for FIC (19.2%) and RVA (0.86%). No purebred animals were found in the RHV breed. The FIC breed was excluded from further analyses due to its low pedigree completeness and low proportion of purebred animals.

**Table 3:** Number and percentage of purebred (> 80% pure breed) for Red Dairy Cattle (RDC), Meuse-Rhine-Yssel (MRY), Finncattle (FIC), Swedish Polled (SKB), Vorderwälder (VWD), Hinterwälder (HWD), Traditional Angler (RVA) and Rotes Höhenvieh (RHV) animals born between 2010 and 2018 within different inbreeding levels

	RDC	MRY	FIC	SKB	VWD	HWD	RVA	RHV
Number of animals	3,180,191	70,005	867	4395	38,234	19	702	187
Number of purebred animals (%)	2,130,052 (67.2)	69,721 (99.6)	167 (19.2)	4204 (95.7)	28,662 (75.0)	7 (36.8)	6 (0.86)	0
Inbreeding class^a^ (%)								
0	19.38	2.53	95.81	12.94	70.79	85.71	83.33	–
0 < $F$ ≤ 6.26	78.75	79.21	1.80	80.28	25.88	0	0	–
6.25 < $F$ ≤ 12.50	1.60	16.42	1.80	4.97	2.45	0	0	–
12.5 < $F$≤ 25.00	0.23	1.75	0.60	1.45	0.77	14.29	16.67	−
> 25.00	0.04	0.19	0	0.36	0.10	0	0	−
Holsteinization (%)	11.3	1.45	10.2	1.6	4.7	4.4	10.8	4.0

^a^Percentage of purebred animals

$$F$$ = inbreeding coefficient

Almost 2.4% (53,885) of the purebred animals in the pedigree had an inbreeding coefficient greater than 6.25%, and all breeds had at least one purebred animal with an inbreeding coefficient greater than 12.5% (Table 3).

We use “Holsteinization” to refer to the proportion of Holstein-ancestry. A mean level of Holsteinization of 13.9% was estimated for the whole population of animals born between 2010 and 2018. High Holsteinization was found in the RDC, FIC and RVA breeds whereas Holsteinization was low in the MRY and SKB breeds (Table 3).

The highest $F$-value (53.2%) was found for a Finnish RDC animal. Among the animals born between 2010 and 2018, 62% had an $F$-value > 0, with RDC, RVA and RHV having the largest proportion of inbred animals, i.e. 75, 84 and 64%, respectively. The mean $F$-value for the population (animals born between 1960 and 2018) was 1.5%, and the two highest $F$-values were observed in the two native German breeds, RHV (4.1%) and RVA (4.2%). The lowest $F$-value was observed in the native HWD breed (0.7%). The relationship within the full pedigree was 1% and ranged from 0.9% for HWD to 4.5% for RHV (Table 2). A mean inbreeding level of 2% was estimated for animals born between 2016 and 2018, with the highest values for MRY (4.64%) and RHV (3.8%). Mean inbreeding values of 2% and 2.3% were estimated for the three RDC, VWD, and RVA breeds, and for SKB, respectively.

**Fig. 2:**
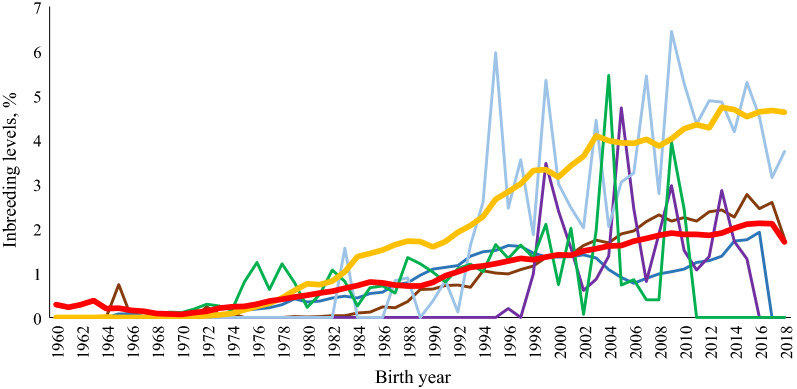
Trend in level of inbreeding for Red Dairy Cattle (RDC; red line), Meuse-Rhine-Yssel (MRY; yellow line), Swedish Polled (SKB; brown line), Vorderwälder (VWD; dark blue line), Hinterwälder (HWD; green line), Traditional Angler (RVA; purple line) and Rotes Höhenvieh (RHV; light blue line), breeds over years

Figure 2 illustrates the mean annual inbreeding by year of birth. The trends in inbreeding were linear for RDC, MRY and SKB, whereas there was a large year-to-year variation for the other breeds. The increase in inbreeding for the entire studied population coincided with an increase in the number of animals of the MRY and RDC breeds (numbers not shown). The highest $F$ value was found for HWD in 2009 (6.44%). In 2018, the highest $F$ value was found for the MRY breed (4.62%), whereas for RDC the inbreeding level dropped from 2.11 to 1.7 between 2017 and 2018.

### Generation intervals

**Fig. 3:**
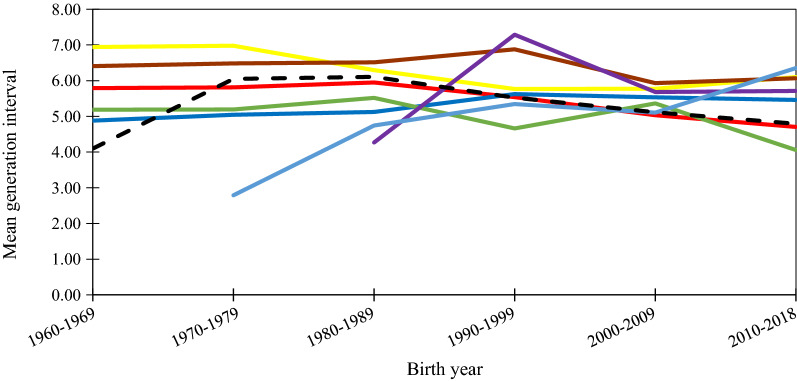
Mean generation interval per 10 years for Red Dairy Cattle (RDC; red line), Meuse-Rhine-Yssel (MRY; yellow line), Swedish Polled (SKB; brown line), Vorderwälder (VWD; dark blue line), Hinterwälder (HWD; green line), Traditional Angler (RVA; purple line) and Rotes Höhenvieh (RHV; light blue line) and ALL breeds (black dotted line) for animals born between 1960 and 2018

Generation intervals varied between the breeds with the shortest interval found for HWD (5.2 years) and the longest for MRY (6.5 years). The mean generation interval for all animals born between 1960 and 2018 was longer (5.5 years) compared to animals born between 2010 and 2018 (4.8 years). Mean generation intervals per 10 years are shown in Fig. 3. For most of the breeds, a small increase in generation interval was found from year 2000 onwards, except for RDC, VWD and SKB for which the generation interval tended to decrease. HWD had a short generation interval during the 1970s because of its small number of animals, i.e. 5.

### Marginal contributions

Table 4 shows the marginal contribution for the top 20 most influential animals born between 1960 and 2018. These animals originated from the Netherlands, Sweden, Denmark, Finland and Norway, and with one exception, were all sires. The most influential animal in the population was a Dutch MRY sire born in 1960. All top 20 most influential animals born between 2010 and 2018 originated from the Nordic countries and explained about 19% of the total marginal contribution (Table 4). The most influential animal in this population was a Danish RDC sire born in 2004. The top 20 most influential animals in either of the populations had a partial inbreeding coefficient lower than 0 and did not disproportionately contribute to the inbreeding level in the red dairy population.

**Table 4:** Top 20 most influential animals born between 1960 and 2018 (to the left) and between 2010 and 2018 (to the right)

Animals born between 1960 and 2018				Animals born between 2010 and 2018			
Breed*Country	Sex	Birth year	Marginal contribution (%)	Breed*Country	Sex	Birth year	Marginal contribution (%)
MRY*NLD	M	1960	3.10	RDC*DNK	M	2004	2.50
RDC*SWE	M	1990	2.41	RDC*SWE	M	2006	1.44
RDC*SWE	M	1991	2.34	RDC*SWE	M	2005	1.35
RDC*NOR	M	1967	2.08	RDC*SWE	M	2004	1.27
MRY*NLD	M	1966	1.85	RDC*FIN	M	2005	1.12
RDC*FIN	M	1960	1.53	RDC*DNK	M	2007	1.10
RDC*SWE	F	1996	1.41	RDC*NOR	M	2004	1.06
RDC*FIN	M	1966	1.40	RDC*NOR	M	2009	0.92
RDC*DNK	M	2004	1.31	RDC*NOR	M	2008	0.88
MRY*NLD	M	1964	1.30	RDC*FIN	M	2004	0.78
MRY*NLD	M	1974	1.28	RDC*SWE	M	2003	0.78
RDC*NOR	M	1987	1.28	RDC*FIN	M	2006	0.72
RDC*NOR	M	1991	1.17	RDC*DNK	M	2004	0.69
RDC*FIN	M	1994	1.02	RDC*NOR	M	2009	0.69
HOL*SWE	M	1964	0.97	RDC*NOR	M	2006	0.59
RDC*FIN	M	1996	0.95	RDC*DNK	M	2006	0.57
MRY*NLD	M	1968	0.92	RDC*FIN	M	2004	0.56
MRY*NLD	M	1978	0.85	RDC*NOR	M	2006	0.56
RDC*SWE	M	1996	0.85	RDC*NOR	M	2009	0.56
RDC*SWE	M	1987	0.82	RDC*FIN	M	2003	0.53

### Gene flow

**Fig. 4:**
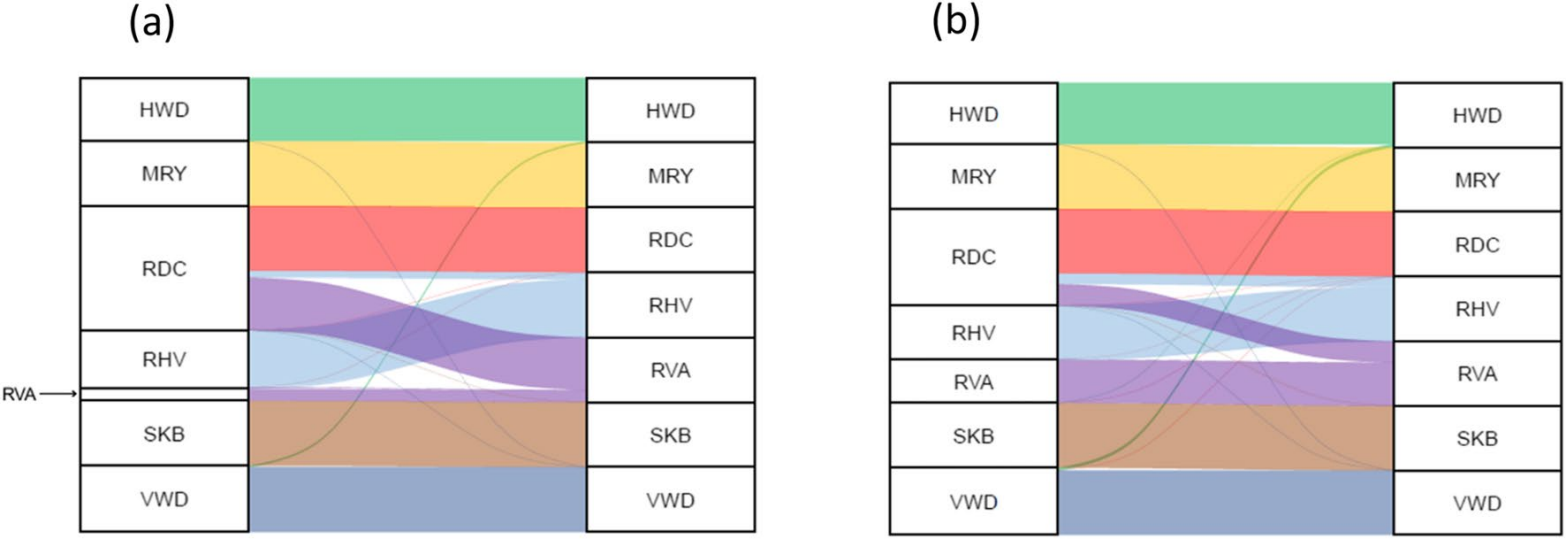
**a**, **b** Alluvial plot of gene flow, between animals born between 1960 and 2018, from sires (left-hand side in **a**) and dams (left-hand side in **b**) of a targeted red dairy breed to an offspring (on the right-hand side in **a** and **b**, respectively) of a targeted red dairy breed. Gene flow colors of the red dairy breeds include, Hinterwälder (HWD) green, Meuse-Rhine-Yssel (MRY) yellow, Red dairy cattle (RDC) red, Rotes Höhenvieh (RHV) light blue, Traditional Angler (RVA) purple, Swedish Polled (SKB) brown and Vorderwälder (VWD) blue

**Fig. 5:**
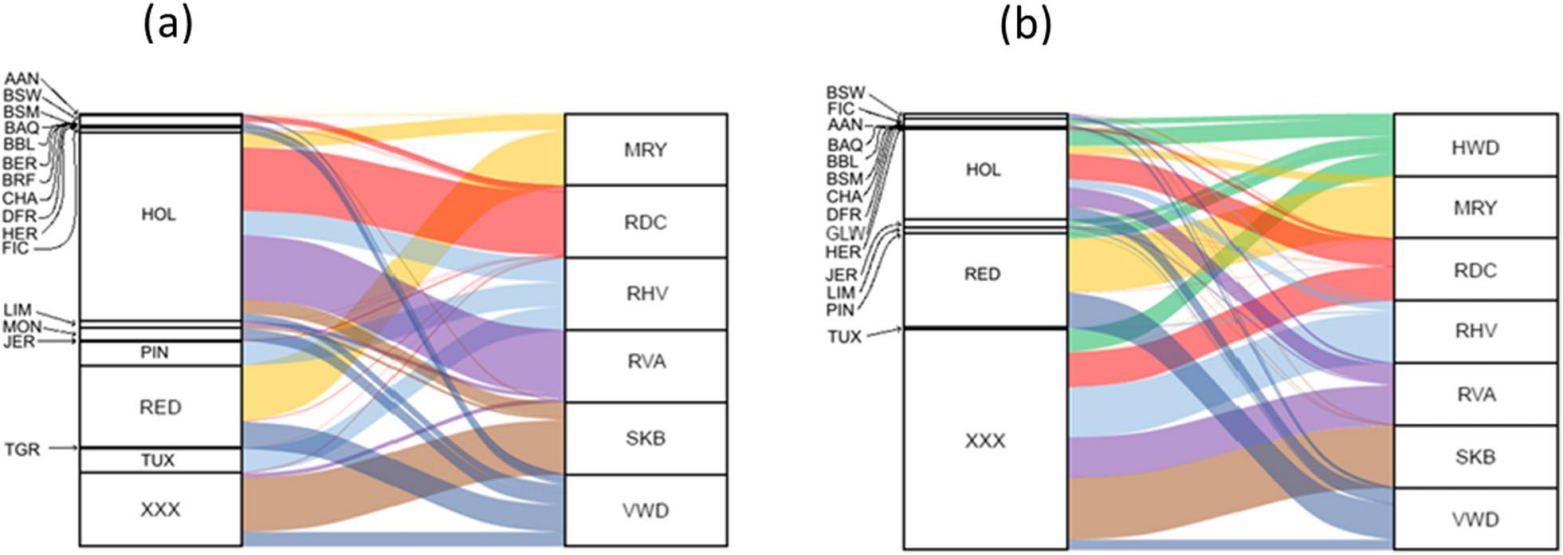
**a**, **b** Alluvial plot of gene flow, between animals born between 1960 and 2018, from sires (left-hand side in **a**) and dams (left-hand side in **b**) of a non-targeted breed to an offspring (on the right-hand side in **a** and **b**, respectively) of a targeted red dairy breed. Gene flow colors of the red dairy breeds include Hinterwälder (HWD) green, Meuse Rhine Yssel (MRY) yellow, Red dairy cattle (RDC) red, Rotes Höhenvieh (RHV) light blue, Traditional Angler (RVA) purple, Swedish Polled (SKB) brown and Vorderwälder (VWD) blue. Non-targeted breeds include Belgian Blue (BBL), Tyrol Grey (TGR), Hereford (HER), Charolais (CHA), Blonde d’Aquitaine (BAQ), Belgian White and Red (BER), Red Holstein (RED), Crossbred (XXX), Holstein (HOL), Brown Swiss (BSW), Limousin (LIM), Aberdeen Angus (AAN), Jersey (JER), Limpburger (LIP), Dutch Friesian (DFR), Simmental/Flekvieh (SIM), Simmental for Beef (BSM), British Friesian (BRF), Montbelliard (MON), Tux (TUX), Pinzgauer (PIN)

Gene flow between breeds is illustrated in Figs. 4 and 5a (sire to offspring) and Fig. 5b (dam to offspring). Both sires and dams of non-targeted breeds and targeted red dairy breeds contributed genes to the targeted red dairy breeds. In the MRY, SKB, RDC, VWD and HWD breeds more than 97% of the sires and more than 91% of the dams were of the same breed as their offspring, while for the RVA and RVH breeds the proportion ranged from 18 to 87% for sire-offspring and from 64 to 80% for dam-offspring. RDC sires and dams were used extensively as parents in both the RVA (77 and 32%) and RVH (10 and 15%) populations.

RVA and RHV descendants were more influenced by other breeds compared to the other targeted red dairy breeds (Figs. 4 and 5). Among the RVA descendants, 18% of the sires and 64% of the dams were of the RVA breed. Among the RHV descendants, 87% of the sires and 80% the dams were of the RHV breed. Both these breeds were mainly influenced by sires and dams of the RDC breed. MRY, RDC, SKB, VWD and HWD descendants had more than 90% of the sires and dams of the same breed. Sires and dams of the Holstein breed were used to breed almost all the targeted red dairy breeds, with one exception: no Holstein sires were identified in the HWD population. Crossbred dams were used as dams in all targeted red dairy breeds, especially in the SKB, RHV and RVA breeds.

Breeding organizations within the countries included in the current study (Denmark, Finland, Germany, Latvia, Lithuania, the Netherlands, Norway, Poland, and Sweden) have used sires from other countries than their own, for breeding purposes. In Germany, the Netherlands, Norway and Poland, more than 93% of their animals were from sires born within the country. In Sweden, Denmark and Finland, the number of animals with sires born within country ranged from 68 to 76%. Only 52% of the Lithuanian animals and 28% of the Latvian animals had sires born within country, with sires from other countries being used.

## Discussion

In contrast to the numerous pedigree analyses on Holstein cattle [35–37] and other dairy or dual-purpose breeds [30, 38, 39], few studies have investigated the genetic diversity of the European red dairy breeds [39–42]. However, none of these studies come close to the size of the current study.

Minor and traditional breeds are in danger of being replaced by higher-yielding modern breeds in highly efficient breeding programs. They are also in danger of losing their genetic integrity due to admixture with higher-yielding breeds. Several of the modern red dairy cattle breeds in Europe produce high-quality milk and in large quantities, display functional and health traits of interest, and show good adaptation to different environments [43]. Proper management of their genetic uniqueness requires a comprehensive knowledge of their pedigrees. By creating a combined pedigree, we assessed the levels and trends of inbreeding and analyzed the pedigree of the red dairy cattle breed populations in Europe. In summary, our findings indicate that, although most of the animals were to some degree born inbred, the current level inbreeding in the major red dairy cattle breeds in Europe remains low, with some exceptions, in particular the MRY and RHV breeds.

The level of inbreeding depends on the breed’s pedigree completeness. In particular, if the fraction of missing parents is large in a pedigree, this can lead to substantial underestimation of the inbreeding level [30, 44]. The mean pedigree completeness in the RDC breed was considerably greater (7.5) than in the other breeds, where the average ECG ranged from 0.6 to 3.4. The main reason of the deeper RDC pedigree is a long history of animal recording in the major RDC subpopulations. In other breeds, incomplete documentation of pedigrees leads to a lower estimated level of pedigree completeness. Incompleteness dictates caution about the overreliance on our data for inbreeding estimations. The level of pedigree completeness increased during the studied years for most of the breeds, which concurs with previous studies and can result from the increased knowledge of breed importance and the improved knowledge about breeding and selection strategies. The FIC breed was excluded from some of the analyses, mainly due to its low pedigree completeness and low proportion of purebred animals, most likely because we did not have access to a complete pedigree for this breed.

Genetic drift and loss of breeds are the most important factors that affect genetic diversity. Uncontrolled inbreeding leads to inbreeding depression which could lead to different negative consequences. In particular, smaller and endangered populations are very exposed to inbreeding. The greatest value of $F$ for any single animal was found in the RDC population (53.2%), which is the breed that had the highest proportion of inbred animals in the pedigree.

The inbreeding coefficient for an individual is very sensitive to the quality of the available pedigree information [30]; thus, absolute inbreeding levels provide less information for comparative purposes than the average rate of inbreeding increase per generation. Nevertheless, the mean level of inbreeding across breeds that are represented in the more recent population was low (2%). The mean $F$ for the RVA and MRY breeds found in the current study was higher than the inbreeding levels previously reported [40, 41]. The mean $F$ was slightly higher for the RDC breed than that reported for the Danish Red [39] which is a synthetic breed, but lower than for the Finnish Ayrshire [42] which is a more closed population. The inbreeding estimate for RDC in this study is an average from different breeds and may not be comparable with inbreeding estimates for individual national breeds. Since the level of inbreeding greatly depends on the depth of the pedigree information, one would expect that the inbreeding coefficients and predictions for homozygosity would be higher for a population for which pedigree recording started earlier. In such cases, it is possible to go further back in the genealogy to identify common ancestors. Therefore, comparison of populations with different base years and different pedigree sizes should be done with caution. If the same year can be used as the beginning of pedigree recording, the obtained estimates of inbreeding will be more comparable among subpopulations.

Since the 1960s, the inbreeding level has steadily increased for the MRY, RDC and SKB breeds (Fig. 2). For the VWD breed, both pedigree completeness and the inbreeding level increased until the mid-1990s and then dropped in 2005, which may be due to the high level of introgressed genes from the Montbéliarde breed after 1995 [45] that are considered unrelated to the VWD genes. The pedigree information for introgressed bulls is much less complete than that for native breeds which may have affected the connectedness within the VWD population in our study. Our study did not include pedigree information for the Montbéliarde breed. Average inbreeding usually increases over time, especially in small and closed populations, where the mating of related individuals cannot be avoided. In the current study, the size of the RVA, RHV and HWD populations was small and the fluctuations of both pedigree completeness and inbreeding level for these breeds may depend on the proportions of animals submitted to the database. In the study of Addo et al. [41], more than 93,000 RVA animals with pedigrees tracing back to 1906 were included whereas in our study, only 1079 RVA animals were submitted with pedigree data tracing back to 1976. For some of the breeds, only a small proportion of the pedigree information was available, which results in low pedigree completeness. For native breeds, small population sizes increase the rate of inbreeding due to genetic drift [46, 47]. Although the MRY breed has a large population size and a low mean $F$, its average level of inbreeding has increased a lot recently, reaching a maximum average $F$ of 4.7% in 2013. This may be due to the decreasing number of MRY animals in recent years and/or to a more intense selection and application of genomic selection. However, controlling the loss in genetic variability of this breed should be addressed.

The parameters derived from the probabilities of gene origin, as described by Boichard et al. [30], are useful to quantify the genetic variability within breeds after a small number of generations, whereas inbreeding coefficients and ${\mathrm{N}}_{e}$ are useful to monitor the genetic variability over a longer time. In animal breeding, maintaining an ${\mathrm{N}}_{e}$ of at least 50 to 100 animals [48] could be used as a guideline to prevent losses in genetic variability. This is by no means a fixed number, but is derived from theoretical arguments that propose that natural selection counteracts inbreeding depression. The estimated ${\mathrm{N}}_{e}$ for the RDC, SKB, VWD and HWD breeds are larger than the above recommended number. In our study, ${\mathrm{N}}_{e}$ for both the VWD and HWD breeds were larger than in a previous study by Hartwig et al. [49], in which the estimated ${\mathrm{N}}_{e}$ were 135 and 165, respectively. In addition, in the study by Hartwig et al. [49], both the number of animals and the years studied differed, which may account for the different results. MRY had an estimated ${\mathrm{N}}_{e}$ of 109, which is just above the recommended number. However, the trend in inbreeding for this breed (Fig. 2) suggests that it may have lost a substantial fraction of its genetic diversity, which agrees with the report by Eynard et al. [40], who argued that the reduced genetic diversity in the MRY breed is a consequence of reduced population size and selection for improved genetic merit. The estimated ${\mathrm{N}}_{e}$ for the RVA breed is near the recommended level, while it is below for the RHV breed. For both these breeds, careful monitoring of the inbreeding rate is necessary to prevent further loss of genetic diversity.

The average generation interval in the MRY, RDC and HWD breeds has decreased since the 1960s (Fig. 3). Decreasing generation interval is a favorable trend in terms of genetic progress, but not necessarily in terms of conservation of genetic diversity, since inbreeding and genetic drift may accumulate faster [50]. With selection programs becoming more intensive and the application of genomic selection, generation intervals will continue to decrease, especially on the bull side [51, 52]. Several studies on Holstein–Friesian cows have found that inbreeding levels have increased rapidly since the implementation of genomic selection in breeding programs [53–55]. Thus, there is a need to implement measures and means for controlling the rate of annual inbreeding, which will help to manage and maintain farm animal genetic resources.

The effective number of founder animals for each population is proportional to the size of the population for all breeds. The same pattern was found in the Irish breeds studied by Mc Parland et al. [29]. In spite of the larger size of the MRY population, the small effective number of founders relative to the other red dairy breeds suggests that this population was derived from a relatively smaller number of animals. The larger effective number of founders relative to the effective number of ancestors across all breeds may indicate past bottlenecks that caused loss of genetic diversity [30] during the development of the European red dairy populations. The ratio between effective number of founders and effective number of ancestors was highest for RDC, VWD and SKB compared to the other breeds. This ratio was higher for RDC, which indicates a narrower bottleneck for this breed than for VWD and SKB. These bottlenecks may have occurred in the early 1970s when inbreeding levels increased.

Founders and individuals near the top of the pedigree are favored in the estimation of marginal contributions. Although the individuals further down the pedigree also make large genetic contributions to the population, these are diluted because they have already been partly accounted for by their ancestors. In our study, a large part of the animals were born in distant years, i.e., near the top of the pedigree and, thus may have a large impact on the estimation of marginal contributions. The uneven number of animals within each breed also influences the estimates of marginal contributions across breeds. Since the sizes of the MRY and RDC populations were the largest, they may have suppressed the contributions of animals in other smaller breeds in the analysis of marginal contributions.

When we analyzed the gene flow among countries, we found that foreign sires and dams were rarely used in the Norwegian population. This is in line with the closed isolated subpopulations observed here, which could be due to the farms being geographically distant in Norway. To test this hypothesis, further studies based on data about the geographical location of the animals are needed.

## Conclusions

We provide the first detailed analysis of the genetic diversity and inbreeding levels of the European red dairy cattle breeds. Based on our results, it is necessary to control the inbreeding level of both the RHV and RVA breeds to prevent larger losses in their genetic diversity. The RDC, VWD, SKB and HWD breeds hold considerably more genetic diversity and are therefore less prone to inbreeding problems. Regarding the MRY breed, because of its decrease in population size, its increasing inbreeding level, and ${\mathrm{N}}_{e}$ being just above the recommended level, it is necessary to implement measures to avoid further loss of genetic diversity. Crossbreeding is one way to increase the genetic variability and may increase the genetic progress. However, given the numerous connections between the various RDC national breeds and the evidence of past introgression from other breeds, this should not be a major concern. The implementation of a joint genomic evaluation across the RDC national breeds would also benefit from the pedigree links among them. Future investigations should aim at genotyping a large number of individuals using high-density genome-wide single nucleotide polymorphism (SNP) arrays or whole-genome sequencing to further analyze the genetic diversity, but at the genetic or genomic level. We suggest that a more detailed genome-based analysis of inbreeding should be performed. For example, by investigating the occurrence of runs-of-homozygosity, we could obtain more information about the inbreeding history, such that inbreeding from a recent ancestor could be distinguished from inbreeding derived from a more distant ancestor.

## Figures and Tables

**Fig. 6:**
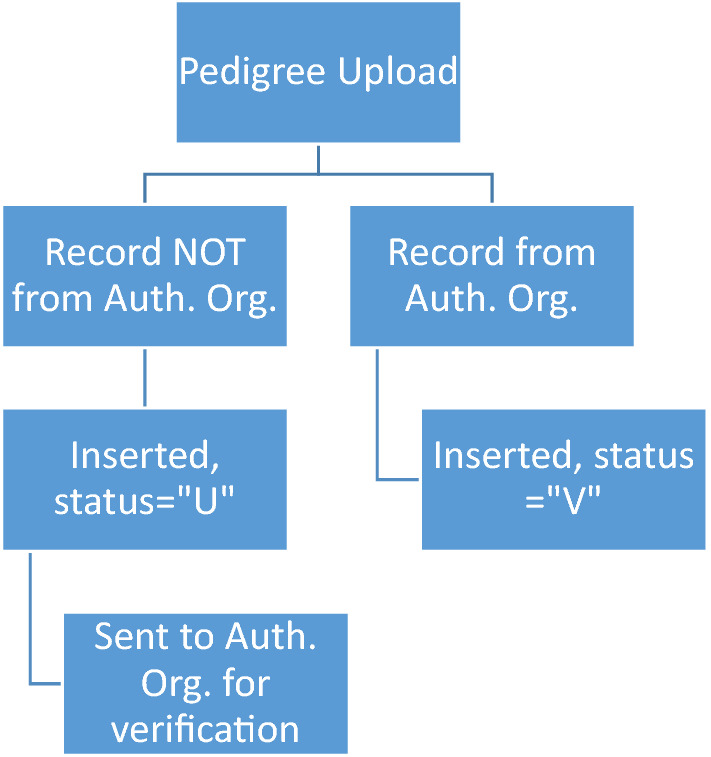
Graphical representation of the pedigree verification inside the Interbull Data Exchange Area database

## Data Availability

The data that support the findings of this study are available from the breeding organizations, but restrictions apply to the availability of these data, which were used under license for the current study, and are not publicly available.

## Appendix

The IDEA pedigree verification process is based on the concept of a so-called “authoritative organization”, an organization considered to be authoritative for a given animal if the country reported in the animal international ID does match with the country the organization belongs to. This means that it is possible to distinguish between domestic and foreign animals included in a pedigree submission: with the domestic animals being those originating from the country to which the organization belongs. Table 5 presents the correspondences between the organizations participating in this study and their authority level. The assumption that underlies an authoritative organization is that pedigree information related to any domestic animals is considered corrected at any given point in time whereas pedigree information related to any foreign animals is not and would require to be confirmed by the relative authoritative organization.

**Table 5:** List of organizations and abbreviations and their authoritative level inside the Interbull Data Exchange Area

Organization	Abbreviation	Authoritative for animals from
Animal Breeders Association of Latvia	ABAL	Latvia (LVA)
CRV	CRV	Netherlands
GENO	GENO	Norway (NOR)
Lithuanian Red Cattle Improvement Association	LRCIA	Lithuania (LTU)
Viking Genetics	VG	Denmark (DNK), Sweden (SWE), Finland (FIN)
Vereinigte Informationssysteme Tierhaltung w.V	VIT	Germany (DEU)
Wroclaw University of Environmental and Life Sciences	WUELS	Poland (POL)
Interbull Centre^a^	ITBC	Other countries

^a^Interbull Centre is acting as the authoritative organization on behalf of animals belonging to countries not included in the study

When a pedigree record is uploaded in IDEA, the system first checks if the organization making the upload is the authoritative one for that animal, and if it is the case the record is inserted in the database and will receive a status code of “V” as verified. In case the organization is not the authoritative one, the record is still inserted but will receive a status code of “U” as unverified. The same pedigree record will also be sent to the relative authoritative organization in order to verify its information. Upon reception of such a request, the authoritative organization will either confirm or correct the pedigree information provided and the animal’s status code will then change from “U” (unverified”) to “V” (verified). Once a pedigree record has a “V” status, no other organizations, beside the authoritative organization, will be able to change its information (Fig. 6).

The verification rate of the pedigree in this study was high (Table 6), i.e. only 1.5% of all uploaded pedigree information appeared as not verified. The highest percentage of unverified pedigree was related to ITBC (Interbull Centre) as it was “acting as” the authoritative organization for animals belonging to countries not included in this study and for which, therefore, a real confirmation of their pedigree information could not be obtained.

**Table 6:** Overview of the verification process including the abbreviated organization name, the number of verified pedigree, number and percentage of unverified pedigree and the total number of pedigree submitted in the database

Organization^a^	Number of verified pedigree	Number of unverified pedigree (*%*)	Total number of pedigree
ABAL	62,533	–	62,533
CRV	2,473,484	–	2,473,484
GENO	2,083,128	9184 *(0.5)*	2,092,312
LRCIA	625,174	22,993 (*3.5*)	648,167
VG	3,332,529	9706 (*0.3*)	3,342,235
VIT	323,755	29,103 (*8.0*)	352,858
WUELS	7062	1300 (*15.0*)	8362
ITBC^b^	30,711	62,741 (*67.0*)	93,452
Overall total	8,938,376	135,027 (*1.5*)	9,073,403

^a^ABAL = Animal Breeders Association of Latvia (Latvia), CRV (the Netherlands), Geno (Norway), LRCIA = Lithuanian Red Cattle Improvement Association (Lithuania), VG = Viking Genetics (Denmark, Sweden, Finland), VIT = Vereinigte Informationssysteme Tierhaltung w.V. (Germany), WUELS = Wroclaw University of Environmental and Life Sciences (Poland) and ITBC = Interbull Centre

^b^Interbull Centre is acting as the authoritative organization on behalf of animals belonging to countries not included in the study
